# *Rickettsia helvetica* in C3H/HeN mice: A model for studying pathogen-host interactions

**DOI:** 10.1016/j.heliyon.2024.e37931

**Published:** 2024-09-14

**Authors:** Apolline Maitre, Lourdes Mateos-Hernandez, Tal Azagi, Angélique Foucault-Simonin, Sabine Rakotobe, Zbigniew Zając, Pavle Banović, Stefania Porcelli, Aurélie Heckmann, Clémence Galon, Hein Sprong, Sara Moutailler, Alejandro Cabezas-Cruz, Andrea C. Fogaça

**Affiliations:** a Anses, INRAE, Ecole Nationale Vétérinaire d’Alfort, UMR BIPAR, Laboratoire de Santé Animale, Maisons-Alfort, France; b INRAE, UR 0045 Laboratoire de Recherches Sur Le Développement de L'Elevage (SELMET-LRDE), 20250, Corte, France; c EA 7310, Laboratoire de Virologie, Université de Corse, Corte, France; d National Institute for Public Health and the Environment, Netherlands; e Department of Biology and Parasitology, Medical University of Lublin, Radziwiłłowska 11 st, 20-080, Lublin, Poland; f Clinic for Lyme Borreliosis and Other Tick-Borne Diseases, Pasteur Institute Novi Sad, 21000 Novi Sad, Serbia; g Department of Microbiology with Parasitology and Immunology, Faculty of Medicine, University of Novi Sad, Novi Sad 21000, Serbia; h Diagnostics and Laboratory Research Task Force, Balkan Association for Vector-Borne Diseases, 21000, Novi Sad, Serbia; i Department of Parasitology, Institute of Biomedical Sciences, University of São Paulo, São Paulo, Brazil

**Keywords:** *Rickettsia helvetica*, Pathogen, Virulence, Tick, Animal models of infection

## Abstract

An infection with the tick-borne *Rickettsia helvetica* has been associated with a broad spectrum of clinical manifestations in humans, but patients are only seldomly reported. Understanding its disease etiology necessitates well-stablished infection models, improving to recognize and diagnose patients with *R. helvetica* infection and facilitating the development of effective control strategies. In this study, we used C3H/HeN mice as a model to establish *R. helvetica* infection, achieving a high infection prevalence (89–100 %). While the liver and the spleen DNA consistently tested positive for infection in all challenged mice, additional infected organs included the kidneys, heart, and the lungs. Notably, a low prevalence of infection was observed in *I. ricinus* nymphs fed on *R. helvetica*-challenged mice. In addition, larvae were refractory to infection, suggesting that ticks exhibit low susceptibility to the pathogen. To the best of our knowledge, this is the first study of an animal model for *R. helvetica* infection. It serves as a valuable tool for advancing research on the interactions among the bacterium and its vertebrate host.

## 1 Introduction

The genus *Rickettsia* (order Rickettsiales, family Rickettsiaceae) comprises gram-negative obligate intracellular bacteria that, according to biological and taxonomic characteristics, are classified in the ancestral group (AG), the typhus group (TG), the transitional group (TrG), or the spotted fever group (SFG). It is noteworthy that all groups, except the ancestral group, include species that cause diseases [1]. Rickettsiae within the SFG (SFGR) are transmitted to vertebrate hosts through the bite of infected ticks. Following transmission, SFGR most probably use monocytes as a vehicle for transport through the host body before invasion of the endothelial cells, where the pathogen extensively proliferates [2]. Both invasion and proliferation inside the host cell depend on different components, including virulence factors that are specific to the bacterial strain [e.g., outer membrane protein B (OmpB) structure, secretion of phospholipase D, hemolysin C, and effectors of Type IV secretion system, etc.] [[3], [4], [5]]. SFGR also express RickA [6] and the surface cell antigen 2 (Sca2) [7], which induce the actin polymerization in host cell and formation of filaments that eject the pathogen through the cytoplasm and cell membrane to adjacent cells or extracellular matrix.

One species within the SFG, *Rickettsia helvetica*, has been associated with development of general and non-specific disease in humans, (i.e., fever, headache, arthralgia, and myalgia) [8] and self-limiting skin lesions [9]. Nevertheless, more severe clinical conditions, such as myocarditis [10], septicemia [11], and meningitis [12] have also been reported, highlighting this bacterium as an emerging human pathogen.

*Ixodes ricinus*, the most abundant tick in Europe, is commonly found infesting humans, acting as the main vector of tick-borne pathogens in the continent, including *R. helvetica* [13,14]. *Rickettsia helvetica* has been also identified in other tick species, such as *Ixodes apronophorus, Ixodes trianguliceps* [15], and *Dermacentor reticulatus* [16,17]. However, the role of these ticks in the epidemiology of the disease remains unreported. *Rickettsia helvetica* has been detected in ticks parasitizing birds in Corsica (France), including migratory species [18]. Moreover, evidence of potential co-feeding transmission of *R. helvetica* in birds (*Parus major*) emphasizes the potential dispersion of this pathogen [19]. Furthermore, vertical transmission is believed to be the primary mechanism for maintaining rickettsial endosymbionts within tick populations [13].

Understanding the interactions among pathogens, the tick vector, and the host are essential to better understand the disease and develop effective control strategies. However, these studies require well-stablished infection models. While previous research demonstrated *R. helvetica* infection in Vero cells (African green monkey kidney cells) [20,21] and L929 cells (murine fibroblasts from connective tissue) [21], attempts to establish *R. helvetica* infection in animal models were unsuccessful [22].

In this study, we successfully infected C3H/HeN mice with the strain DK2 of *R. helvetica*, marking the first report on the establishment of an animal model for *R. helvetica* infection. Of note, *I. ricinus* was only partially susceptible to infection after an acquisition feeding on infected mice, highlighting the low susceptibility of larvae and nymphs of this tick species to infection through feeding. This model is now available for additional studies on *R. helvetica*-vertebrate host interactions.

## 2 Results

### 2.1 Initial experiment: single mouse challenge

In the first experiment, one C3H/HeN mouse was challenged with the *R. helvetica* strain DK2, using the same route of infection previously established for *R. rickettsii* [23,24]. The gDNA extracted from the mouse organs on the fifth DPI served as a template for conventional PCR. The expected 147 bp amplicon was observed in the liver, the spleen, kidneys, and the lungs (Fig. S1).

### 2.2 Second experiment: confirmation and extension

**Fig. 1:**
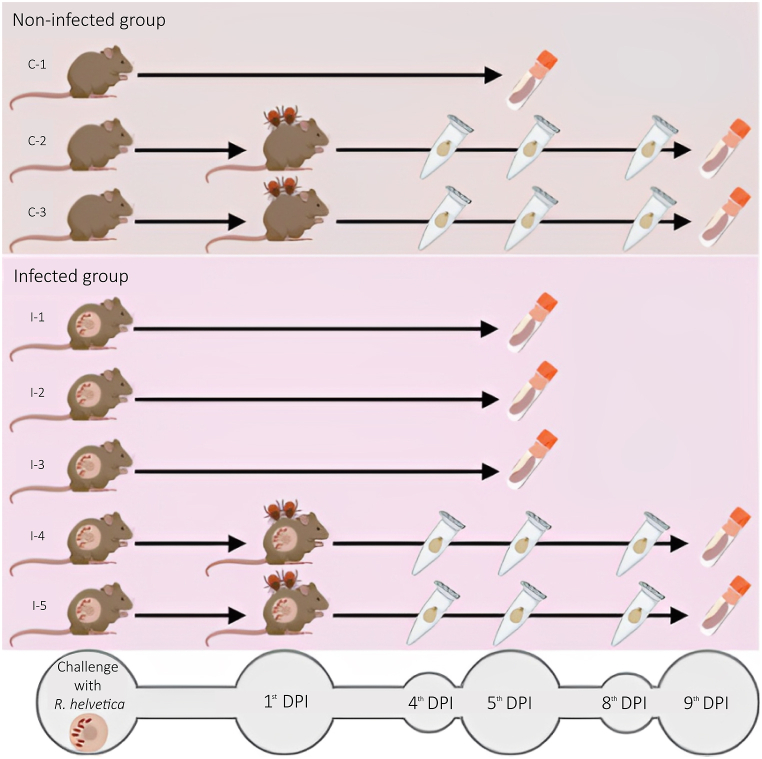
Schematic representation of mouse groups in experiments. Five naïve mice were challenged with *R. helvetica* (infected group), while three animals were maintained as a control (noninfected group). On the fifth day post-infection (DPI), one noninfected and two *R. helvetica*-infected mice were euthanized for organ collection. On the first DPI, two noninfected and two infected mice were infested with *I. ricinus* nymphs. Fully engorged nymphs were collected daily from the fourth to the eighth DPI. The mice that served as hosts for the ticks were euthanized on the nineth DPI for organ collection. Figure created by BioRender (www.biorender.com).

**Fig. 2:**
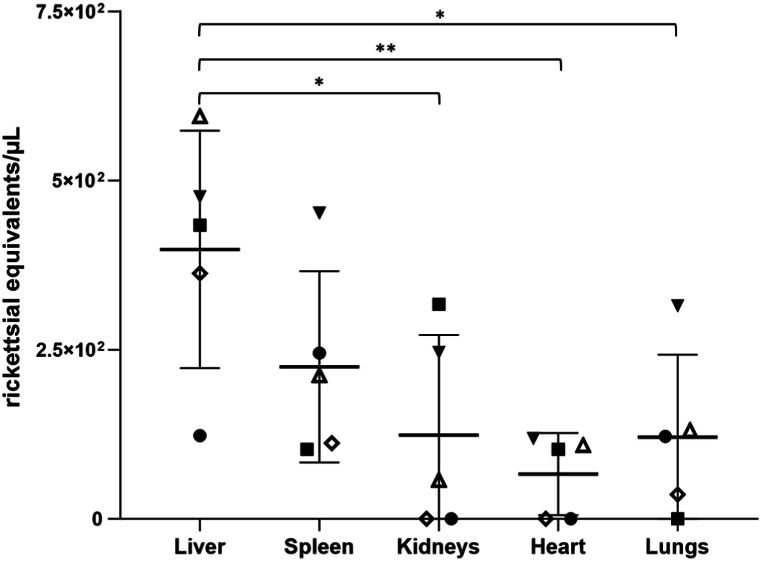
***Rickettsia helvetica* quantification in mouse organs.** The number of genome equivalents of *R. helvetica* was determined by qPCR using specific primers for the single-copy gene *gltA* of *Rickettsia* sp. Solid symbols represent the organs of mice dissected on the fifth DPI and empty symbols those dissected on the nineth DPI. Horizontal lines represent the median of rickettsial equivalents of all animals in a specific organ. ∗*p* < 0.05; ∗∗p < 0.01 (Mann-Whitney test).

Once the adequacy of the infection route and the inoculum size were confirmed, a second experiment was conducted. Five mice were challenged with *R. helvetica*, while three mice served as controls. On the fifth DPI, three challenged and one control mice were euthanized for organ collection (Fig. 1). The gDNA extracted from the organs was used for qPCR to detect and quantify *R. helvetica* (Table 1 and Fig. 2). The liver and spleen of all infected mice were positive, along with the kidneys, the heart and the lungs of two of them (Table 1 and Fig. 2). Subsequently, two challenged and two control mice were infested with *I. ricinus* nymphs on the first DPI and euthanized for organ collection on the ninth DPI (Fig. 1). The liver, spleen and lungs of both challenged mice were positive for *R. helvetica* (Table 1). Of note, rickettsiae were not detected in any mice of the control group (Table 1). Despite a 100 % prevalence of infection, classic murine signs of spotted fever were not observed, and no organ alterations were visible during dissection.

**Table 1:** Molecular detection of *R. helvetica* in organs of C3H/HeN mice from infected and noninfected groups.

Organ	Infected Group						Noninfected group			
	5th DPI			9th DPI		Total	5th DPI	9th DPI		Total
	I-1	I-2	I-3	I-4	I-5		C-1	C-2	C-3	
**Liver**	+	+	+	+	+	100 % (5/5)	ND	ND	ND	0 % (0/3)
**Spleen**	+	+	+	+	+	100 % (5/5)	ND	ND	ND	0 % (0/3)
**Kidneys**	ND	+	+	ND	+	60 % (3/5)	ND	ND	ND	0 % (0/3)
**Heart**	ND	+	+	ND	+	60 % (3/5)	ND	ND	ND	0 % (0/3)
**Lungs**	+	ND	+	+	+	80 % (4/5)	ND	ND	ND	0 % (0/3)

ND: Not detected.

### 2.3 Third experiment: test of larvae infection

A third experiment was conducted to test the infection of *R. helvetica* on *I. ricinus* larvae. Similarly to nymph infection, larvae were fed on mice challenged or not with *R. helvetica*. The spleen of seven from nine mice inoculated with *R. helvetica* was positive for infection (Fig. S2). Of note, rickettsiae were not detected in mice of the control group (Fig. S2). Despite an 89 % prevalence of infection, none mouse exhibited the classic murine signs of spotted fever and no organ alterations were visible during dissection, as observed in the previous experiments.

### 2.4 Molecular confirmation

To further confirm infection, gDNA extracted from the mouse spleen and *R. helvetica*-infected Vero cells was used for conventional PCR with primers targeting either the gene *gltA* or *rickA* of *Rickettsia* (Table S1). The amplicons for *gltA* (Fig. S3A) and *rickA* (Fig. S3B) were observed in all reactions using gDNA from infected mice and Vero cells, but not in those using gDNA from control mice. Sequences obtained in the current study are available in Supplementary File S1.

**Fig. 3:**
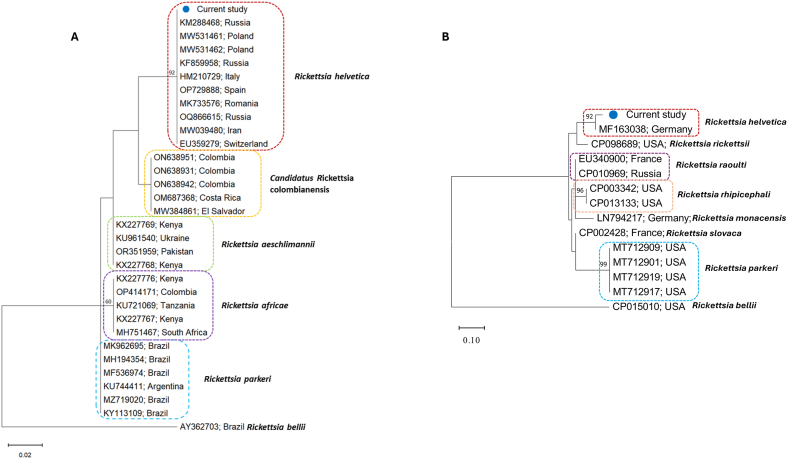
**Phylogeny of Spotted Fever Group Rickettsia.** The evolutionary history of SFGR was based on *gltA* (**A**) and *rickA* (**B**) genes and inferred by using the Maximum Likelihood method and the Tamura 3-parameter model (T92) and Kimura 2-paramenter model, respectively. The analysis contains sequences identified in the current study (marked with blue dot and available in Supplementary File S1) and those collected from GenBank database. Accession numbers and country of origin are given. Bootstrap values are represented as percentage of internal branches (1000 replicates), and values lower than 60 are hidden. The tree is drawn to scale, with branch lengths measured in the number of substitutions per site. *Rickettsia bellii* sequence AY362703 was used to root the tree for *gltA*, while *R. bellii* sequence CP015010 was applied to root the *rickA* tree. (For interpretation of the references to colour in this figure legend, the reader is referred to the Web version of this article.)

Phylogenetic analysis of the *gltA* and *rickA* sequences obtained in the current study confirmed the species affiliation to *R. helvetica* (Fig. 3). Derived consensus *gltA* sequence clustered with others previously reported from European countries including Poland, Romania, Ukraine, Italy, Switzerland and also from Asia, including Iran and Russia (Fig. 3A). The analyzed sequences were characterized by low genetic diversity. As expected, within the study population (*R. helvetica* infecting Vero cells or mice), only one haplotype was identified within *gltA* gene (Fig. 3A), confirming the consistency of the *R. helvetica* strain across the infectious progress. In addition, examined *rickA* sequence, also confirmed species affiliation to *R. helvetica* (Fig. 3B).

### 2.5 Tick infection assessment

A total of 17 fully engorged nymphs were obtained from the *R. helvetica*-infected mice group, and 23 from the control group. *Rickettsia helvetica* was detected in one tick fed on one mouse of the infected group (Table 2). Additionally, seven engorged nymphs from the control group were randomly selected for qPCR analysis, and all were negative for *R. helvetica* infection (Table 2). In a third infection experiment, a total of 13 pools of 10 fully engorged larvae each were obtained from the *R. helvetica*-infected mice and 4 pools from noninfected mice. *Rickettsia helvetica* was not detected in any of the larvae pools (Table 3).

**Table 2:** Number of engorged nymphs collected from infected and noninfected C3H/HeN mice and molecular detection of *R. helvetica*.

	Infected group		Noninfected group	
Dropping day	I-4	I-5	C-2	C-3
4th DPI	0	2	1	3
5th DPI	4	2	5	5
6th DPI	4	1	5	3
7th DPI	0	3	0	0
8th DPI	1	0	0	1
**Prevalence of infected ticks**	0 % (0/9)	12.5 % (1/8)	0 % (0/11)	0 % (0/12)

**Table 3:** Number of pool of engorged larvae collected from infected and noninfected C3H/HeN mice and molecular detection of *R. helvetica*.

	Infected group							Noninfected group	
Dropping day	I-6	I-7	I-8	I-10	I-11	I-13	I-14	C-4	C-5
4th DPI	2	2	1	2	1	1	2	2	0
5th DPI	1	0	0	0	0	0	1	0	2
**Prevalence of infected ticks**	0 % (0/3)	0 % (0/2)	0 % (0/1)	0 % (0/2)	0 % (0/1)	0 % (0/1)	0 % (0/3)	0 % (0/2)	0 % (0/2)

## 3 Discussion

The C3H/HeN murine model, well-established for studying interactions among vertebrate hosts, ticks, and tick-borne pathogens [[23], [24], [25], [26]], and the most adequate murine model for SFGR, as the endothelial cells are the main infection target [27], was the focus of our investigation into the susceptibility of these mice to *R. helvetica*. Remarkably, 89 %–100 % prevalence of infection was achieved, contrasting with a previous report indicating Balb/c and C3H/HeN mice as non-susceptible to *R. helvetica* [22]. In the mentioned study, none Balb/c mouse was positive for infection, and only 20 % (1/5) of C3H/HeN mice became infected after *R. helvetica* challenge alone or in association with *Borrelia afzelii* [22]. Discrepancies in outcomes may arise from methodological variations, such as the *R. helvetica* strain used (isolated from an *I. ricinus* specimen from Slovakia vs. the strain DK2 from the Netherlands), the number of rickettsiae in the inoculum (8 x 10^4^ vs. 4 x 10^9^ rickettsiae per mouse), the inoculation route [intraperitoneal and subcutaneous vs. intravenous (retro-orbital)], and the time point analyzed after infection (14th DPI vs. 5th and 9th DPI). Of note, a previous attempts of our research group using a smaller number of rickettsiae in the inoculum (i.e., 1 x 10^7^ - 1 x 10^9^ rickettsiae per mouse) and the intraperitoneal infection route resulted in the absence of infection (data not shown). Therefore, introducing a pathogen intravenously seems to be key to bypass dendritic (antigen-presenting) cells in peritoneum and mucosa, making the innate immune response less efficient in the initial moments of infection [28].

In comparison to infection with *R. rickettsii*, known to induce spotted fever signs (including ruffled fur, shallowed respiration, hunched posture, decreased activity, limited motility or prostration) and mortality in C3H/HeN mice within five days [23,24], mice challenged with *R. helvetica*, even at a two-orders-of-magnitude higher dose (4.0 x 10^9^ vs 10^7^ in *R. rickettsii*), did not exhibit disease signs or mortality. In addition, no apparent organ alteration was observed upon gross examination. It was previously shown that the severity of infection in mice [23] and in Guinea pigs (*Cavia porcellus*) [29] varies according to the strain of *R. rickettsii*. Therefore, further investigation on the disease evolution in C3H/HeN mice challenged with different *R. helvetica* strains are warranted. In addition, the murine model established by the current study can also be used for comparative analyses on host responses to infection with *R. helvetica* and more virulent rickettsial species.

Concerning tick infection, a low number of engorged *I. ricinus* nymphs was obtained after feeding on C3H/HeN, whether infected with *R. helvetica* or not. *Rickettsia helvetica* was detected in only one from 17 nymphs fed on infected mice, yielding 5.9 % infection prevalence. Moreover, none of the pools of larvae fed on infected mice were positive for *R. helvetica*. This aligns with the prevalence observed in the experimental infection of *Amblyomma sculptum* ticks with *R. rickettsii*, which oscillates around 10 % [30,31]. A slightly higher susceptibility is observed when ticks are exposed to autochthonous *R. rickettsii* [32,33]. The limited susceptibility of *A. sculptum* to *R. rickettsii* is attributed to the upregulation of immune factors in the tick midgut upon infection [30]. It was previously shown that the midgut of *I. ricinus* rapidly clear both gram-positive (*Micrococcus luteus*) and gram-negative (*Pantoea* spp.) bacteria ingested by a capillary feeding, possibly due to the presence of antimicrobial factors [34]. Additional studies are warranted to assess the effect of *R. helvetica* on the expression of immune-related genes of *I. ricinus*, which may be involved in infection control. The low prevalence of *R. helvetica*-infected ticks after an experimental infection is also in consonance with the prevalence observed in field-collected ticks [[35], [36], [37], [38], [39], [40], [41], [42]]. For instance, among questing ticks, 1.8 % were positive for *R. helvetica* in Poland and Bulgaria [35], 4.14 % in Denmark [36], 5.1 % in Siberia [37], and only 0.04 % in Scotland [38], while lightly higher prevalence values were reported in Sweden (22 %) [39] and France (14–27 %) [40]. Among ticks collected from wild and/or domestic animals, 6 % were detected to be infected in Corsica (France) [41] and 11.5–14.4 % in the Netherlands [42]. Previous studies have shown that the presence of *R. helvetica* in *I. ricinus* ticks alters tick microbiota, reducing bacterial alpha-diversity [43]. Furthermore, *R. helvetica* shows a positive correlation with *Candidatus* Midichloria mitochondrii [43], which influences the feeding fitness of *I. ricinus* larvae [44]. Therefore, additional studies are warranted to understand the effects of endosymbionts on the acquisition of *R. helvetica* by ticks.

In conclusion, the murine model of *R. helvetica* infection established in this study provides a valuable platform for further research into the disease mechanisms and the development of effective control strategies, including vaccines. For example, the vaccination of C3H/HeN mice with rickettsial proteins has previously been used to assess protection against *R. rickettsii* [[45], [46], [47], [48]]. Given the diverse clinical manifestations of *R. helvetica* infection in humans, advancing our understanding of its pathogenesis and developing effective control measures are of utmost importance.

## 4 Material and methods

### Ethics approval

Procedures with living animals were performed at the Animal Facility of the Laboratory for Animal Health of the French Agency for Food, Environmental and Occupational Health & Safety (ANSES), Maisons-Alfort, France, according to the French and International Guiding Principles for Biomedical Research Involving Animals (2012). The procedures were reviewed and approved by the Ethics Committee (ComEth, Anses/ENVA/UPEC), with permit number E9404608.

### *4.1* *Rickettsia helvetica* cultivation

The *R. helvetica* strain (DK2) was isolated from a male *Ixodes ricinus* collected in the costal dunes of the Netherlands (coordinates 52.4347; 4.6258), in 2018. For cultivation, rickettsiae were propagated in Vero cells (ATCCTM CCL-81TM, Manassas, VA, United States). After seven days at 32 °C, infected cells were collected, centrifuged at 175×*g* for 5 min, and then used to infect additional cells. Subsequent to an additional seven extra days at 32 °C, infected cells were harvested and suspended in Minimal Essential Medium (MEM, ThermoFisher Scientific, USA). The medium was supplemented with 2 mM L-glutamine and 10 % heat-inactivated fetal bovine (FBS) serum, and containing 10 % dimethyl sulfoxide (DMSO). An aliquot of infected Vero cells was used for genomic DNA (gDNA) extraction and quantification of rickettsiae in the inoculum by qPCR, as described below. The *R. helvetica* inoculum was stored in liquid nitrogen (N_2_) before use.

### 4.2 Ticks

The *I. ricinus* colony (French strain) was maintained at the ANSES animal facility in Maisons-Alfort, France. Larvae, nymphs and adults were fed on 11-week-old New Zealand white rabbits (*Oryctolagus cuniculus*, Charles River strain code 052), purchased from Charles River Laboratories (Miserey, France). The rabbits were housed in standard cages with unrestricted access to food and water. The room temperature (RT) was maintained at a controlled range of 20–23 °C, and a 12-h light:12-h dark photoperiod was followed. Throughout the entire experimental process, the health and behavior of the animals were carefully monitored twice daily by experienced technicians. During off-host phases, ticks were maintained in an incubator set at 22 °C with a relative humidity (RH) exceeding 97 %, following a 12-h light-dark cycle. Alternatively, larvae of *I. ricinus* were purchased from IS Insect Services GmbH (Germany).

### 4.3 Mouse infection and tick infestation

Male C3H/HeN mice (*Mus musculus*), 7-8-week-old, were purchased from Charles River Laboratories. For inoculum preparation, initially, Vero cells infected with *R. helvetica* were centrifuged at 3000×*g* for 5 min. The pellet was resuspended in sterile phosphate buffered saline (PBS) and subjected to three heating cycles at 37 °C, followed by 30 s (s) in liquid N_2_ to release rickettsiae.

In the first preliminary experiment, one mouse (I-0) was anesthetized with isoflurane (Zoetis, New Jersey, USA) and inoculated with 4.0 x 10^9^ equivalent genomes of *R. helvetica* (see below) in 50 μL of sterile PBS via the retro-orbital sinus, using an insulin 26-gauge needle and 1 mL syringe (BD Plastipak; Becton, Dickinson and Company, New Jersey, USA). On the fifth day post-infection (DPI), the mouse was euthanized with CO_2_, and organs (spleen, kidneys, liver, heart, lungs, and skin) were collected and stored at −80 °C.

In a second experiment (see Fig. 1), five mice (I-1, I-2, I-3, I-4, and I-5) were anesthetized and infected as described above. Three naïve mice served as controls (C-1, C-2, and C-3). On the fifth DPI, three challenged mice (I-1, I-2, and I-3) and one control (C-1) mouse were euthanized for organ collection. On the first DPI, two of the five *R. helvetica*-challenged mice (I-4 and I-5), as well as two control mice (C-2 and C-3), were infested with *I. ricinus* nymphs (30 nymphs/mouse). Nymphs were placed in EVA-foam (Cosplay Shop, Belgium) capsules glued to the shaved backs of the animals, as previously described [49]. Fully engorged nymphs were collected daily from the fourth to the eighth DPI, washed once in 70 % ethanol and once in sterile PBS, and stored at −80 °C. In a third experiment, nine mice (I-6, I-7, I-8, I-9, I-10, I-11, I-12, I-13, and I-14) were infected with *R. helvetica* as described above and used as hosts for larvae of *I. ricinus*. As a control, larvae were fed on two noninfected mice (C-4 and C-5). Fully engorged larvae were collected from three to four DPI, washed and stored as detailed for nymphs. Nine pools of 10 larvae each were obtained from infected mice and four pools from control mice.

The mice hosting the ticks were euthanized on the nineth DPI for organ collection. During the experiments, we monitored disease signs in mice daily, including ruffled fur, shallow breathing, hunched posture, and decreased activity, as outlined by Ref. [23].

### 4.4 Genomic DNA extraction and absolute quantification of *R. helvetica*

To extract gDNA from mouse organs (∼20 mg per organ) and fully engorged *I. ricinus* nymphs and larvae (pools of 10 specimens each), homogenization was performed with sterilized metal beads using a Precellys24 Dual homogenizer (Bertin technologies, France). Three cycles at 5500 rpm for 20 s were applied. gDNA from the homogenized samples or *R. helvetica*-infected Vero cells was then extracted using the NucleoSpin® Tissue kit (Macherey-Nagel, Germany), according to the manufacturer's instructions.

The resulting gDNA served as a template in quantitative PCR (qPCR) using specific primers for the citrate synthase (*gltA*) gene and the LightCycler 480 SYBRGreen kit (Roche, Switzerland; for qPCR analyses). The qPCR analyses were carried out in a LightCycler® 480 system II (Roche, Switzerland) with the following thermocycler program: 5 min at 95 °C followed by 45 cycles of 10 s at 95 °C, 15 s at 55 °C, and 15 s at 72 °C. The quantitation cycle (C_q_) of each sample was determined by the LightCycler 480 software (Roche, Switzerland) and compared with the C_q_ of a standard curve constructed with serial dilutions of the *gltA* amplicon*,* as detailed by Ref. [50]. The C_q_ values were subsequently used to calculate the number of genomic equivalents of rickettsiae per μL of gDNA sample. As a control, reactions were carried out in the absence of the gDNA. All samples were analyzed in technical triplicates.

### 4.5 Nucleotide sequencing

The gDNA extracted from both the spleen of mice and the inoculum of *R. helvetica* in Vero cells served as the template in conventional PCR, with the specific primers for *gltA* CS78 and CS283 [51] or *rickA* [52] (Table S1). The resulting amplicons were separated by electrophoresis on a 2 % agarose gel with ethidium bromide (final concentration of 0.5 μg/mL) and visualized using a transilluminator. Subsequently, selected amplicons were excised from the gel and purified using the NucleoSpin® Gel and PCR Clean-up kit (Macherey Nagel). Nucleotide sequencing was performed by Eurofins (France).

### 4.6 Phylogenetic analysis

The sequences obtained in the current study were analyzed using the Basic Local Alignment Search Tool (BLAST; https://blast.ncbi.nlm.nih.gov/Blast.cgi, accessed on January 23, 2024). In the next step, sequences showing similarity were aligned using the MUSCLE algorithm in MEGA 11. Based on the lowest Bayesian Information Criterion and corrected Akaike Information Criterion, the Tamura 3-parameter model (T92) and Kimura 2-parameter (K2) models were used to construct phylogenetic trees of the Rickettsia *gltA* and *rickA* genes, respectively. The sequences representing the *rickA* gene were obtained by trimming the corresponding nucleotides from the complete genomes of the particular *Rickettsia* species. The evolutionary history was inferred by using the Maximum Likelihood (ML) method with complete deletion option, bootstrap set at 1000 and analyzed in MEGA 11 [53].

In addition, to determine genetic diversity of sequences clustered together with those obtained in the current study, i.e., *R. helvetica gltA* were grouped into haplotypes (genotypes) using the DnaSP software (Universitat de Barcelona, Spain, http://www.ub.edu/dnasp, accessed on January 23, 2024).

### 4.7 Statistical analysis

The statistical analysis of rickettsial loads in mouse organs inoculated with *R. helvetica* was performed using Mann-Whitney test in GraphPad Prism version 8.0 for Windows (GraphPad Software, USA). Differences between two organs were considered statistically significant if the *p*-value was <0.05.

## Funding

This work was supported by the French Government's Investissement d’Avenir program, Laboratoire d’Excellence “Integrative Biology of Emerging Infectious Diseases” (Grant ANR–10–LABX–62–IBEID). ACF received a research fellowship from the São Paulo Research Foundation (FAPESP; grant 2022/08257–0) and a research productivity fellowship from the National Council for Scientific and Technological Development (CNPq; grant 309733/2018–9). AM was supported by the ‘Collectivitéde Corse’, grant: ‘Formations superieures’ (SGCE-RAPPORT No. 0300).

## Data availability

The nucleotide sequences obtained in this study are available in Supplementary File S1, which can be accessed along with the article.

## CRediT authorship contribution statement

**Apolline Maitre:** Writing – review & editing, Writing – original draft, Visualization, Investigation. **Lourdes Mateos-Hernandez:** Writing – review & editing, Investigation. **Tal Azagi:** Writing – review & editing, Methodology, Investigation. **Angélique Foucault-Simonin:** Writing – review & editing, Investigation. **Sabine Rakotobe:** Writing – review & editing, Investigation. **Zbigniew Zając:** Writing – review & editing, Writing – original draft, Visualization, Formal analysis, Data curation. **Pavle Banović:** Writing – review & editing, Writing – original draft. **Stefania Porcelli:** Writing – review & editing, Investigation. **Aurélie Heckmann:** Writing – review & editing, Investigation. **Clémence Galon:** Writing – review & editing, Investigation. **Hein Sprong:** Writing – review & editing, Supervision, Resources, Investigation, Conceptualization. **Sara Moutailler:** Writing – review & editing, Supervision, Resources, Conceptualization. **Alejandro Cabezas-Cruz:** Writing – review & editing, Writing – original draft, Supervision, Resources, Conceptualization. **Andrea C. Fogaça:** Writing – review & editing, Writing – original draft, Visualization, Supervision, Methodology, Investigation, Formal analysis, Data curation, Conceptualization.

## Declaration of competing interest

The authors declare that they have no known competing financial interests or personal relationships that could have appeared to influence the work reported in this paper.

## Appendix A Supplementary data

Multimedia component 1 Agarose gel electrophoresis of PCR amplicons from *R. helvetica*-infected mouse tissues. The gDNA extracted from the liver (Li), spleen (Sp), Kidneys (Kd), heart (H), lungs (Lu), and skin (Sk) of a *R. helvetica*-infected mouse (I-0) was used as template in conventional PCR using the specific primers for *gltA*-PCR (Table S1). Amplicons were separated on a 2% agarose gel electrophoresis stained with ethidium bromide and visualized under UV light. DNA marker size (bp) is shown. PC (positive control): fragment of *gltA* clone in a plasmid; NT: non template Multimedia component 1

Multimedia component 2 PCR Analysis of *R. helvetica* infection in mice spleens. The gDNA extracted from the spleen of noninfected (C-4 and C-5) and *R. helvetica*-infected mice (I-6, I-7, I-8, I-9, I-10, I-11, I-12, and I-14), was used as template in conventional PCR using the specific primers for *gltA*-PCR (Table S1). Amplicons were separated on a 2% agarose gel electrophoresis stained with ethidium bromide and visualized under UV light. DNA marker size (bp) is shown. RH: inoculum of *R. helvetica* in Vero cells; NT: non-template control Multimedia component 2

Multimedia component 3 PCR detection of *gltA* and *rickA* genes in *R. helvetica*-infected mice and Vero cells. The gDNA extracted from the spleen of noninfected (C-1, C-2, and C-3) and *R. helvetica*-infected mice (I-0, I-1, I-2, I-3, I-4, and I-5), as well as infected Vero cells, was used as template in conventional PCR using the specific primers for either *gltA* or *rickA* sequencing (Table S1). Amplicons for *gltA* (**A**) or *rickA* (**B**) were separated on a 2% agarose gel electrophoresis stained with ethidium bromide and visualized under UV light. DNA marker size (bp) is shown. RH: inoculum of *R. helvetica* in Vero cells; NT: non-template control Multimedia component 3
