# Supplementary material for: Rickettsia helvetica in C3H/HeN mice: A model for studying pathogen-host interactions
Source: Heliyon. 2024 Sep 14;10(18):e37931. doi: 10.1016/j.heliyon.2024.e37931 (PMC11422568; doi:10.1016/j.heliyon.2024.e37931)

**Supplementary Figure S1.** Agarose gel electrophoresis of PCR amplicons from *R. helvetica*-infected mouse tissues.


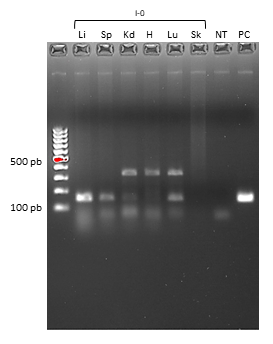

Supplement: Multimedia component 1 — Agarose gel electrophoresis of PCR amplicons from R. helvetica-infected mouse tissues. The gDNA extracted from the liver (Li), spleen (Sp), Kidneys (Kd), heart (H), lungs (Lu), and skin (Sk) of a R. helvetica-infected mouse (I-0) was used as template in conventional PCR using the specific primers for gltA-PCR (Table S1). Amplicons were separated on a 2% agarose gel electrophoresis stained with ethidium bromide and visualized under UV light. DNA marker size (bp) is shown. PC (positive control): fragment of gltA clone in a plasmid; NT: non template [file mmc1.docx]
