# Supplementary figures and images for: Rickettsia helvetica in C3H/HeN mice: A model for studying pathogen-host interactions
Source: Heliyon. 2024 Sep 14;10(18):e37931. doi: 10.1016/j.heliyon.2024.e37931 (PMC11422568; doi:10.1016/j.heliyon.2024.e37931)

**Supplementary Figure S2.** PCR Analysis of *R. helvetica* infection in mice spleens.


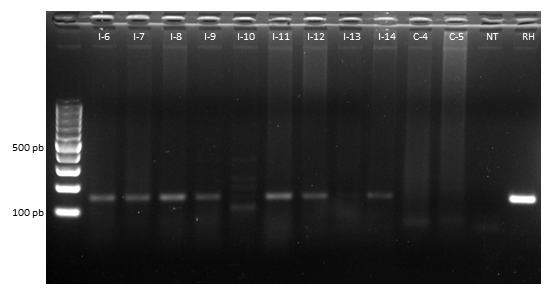

Supplement: Multimedia component 2 — PCR Analysis of R. helvetica infection in mice spleens. The gDNA extracted from the spleen of noninfected (C-4 and C-5) and R. helvetica-infected mice (I-6, I-7, I-8, I-9, I-10, I-11, I-12, and I-14), was used as template in conventional PCR using the specific primers for gltA-PCR (Table S1). Amplicons were separated on a 2% agarose gel electrophoresis stained with ethidium bromide and visualized under UV light. DNA marker size (bp) is shown. RH: inoculum of R. helvetica in Vero cells; NT: non-template control [file mmc2.docx]
