# Supplementary material for: Rickettsia helvetica in C3H/HeN mice: A model for studying pathogen-host interactions
Source: Heliyon. 2024 Sep 14;10(18):e37931. doi: 10.1016/j.heliyon.2024.e37931 (PMC11422568; doi:10.1016/j.heliyon.2024.e37931)
Supplement: Multimedia component 3 — PCR detection of gltA and rickA genes in R. helvetica-infected mice and Vero cells. The gDNA extracted from the spleen of noninfected (C-1, C-2, and C-3) and R. helvetica-infected mice (I-0, I-1, I-2, I-3, I-4, and I-5), as well as infected Vero cells, was used as template in conventional PCR using the specific primers for either gltA or rickA sequencing (Table S1). Amplicons for gltA (A) or rickA (B) were separated on a 2% agarose gel electrophoresis stained with ethidium bromide and visualized under UV light. DNA marker size (bp) is shown. RH: inoculum of R. helvetica in Vero cells; NT: non-template control [file mmc3.docx]

**Supplementary Figure S3.** PCR detection of *gltA* and *rickA* genes in *R. helvetica*-infected mice and Vero cells.


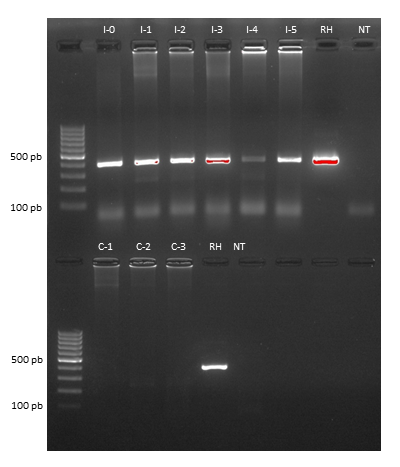


**A**


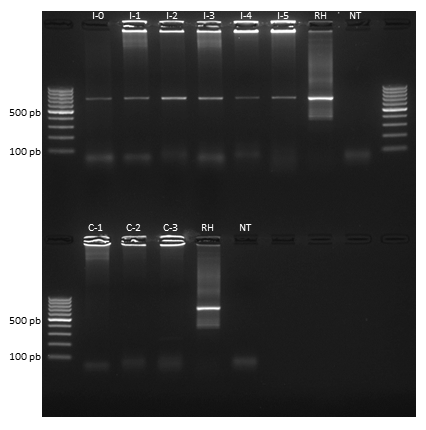


**B**
