# Supplementary material for: Rickettsia helvetica in C3H/HeN mice: A model for studying pathogen-host interactions
Source: Heliyon. 2024 Sep 14;10(18):e37931. doi: 10.1016/j.heliyon.2024.e37931 (PMC11422568; doi:10.1016/j.heliyon.2024.e37931)
Supplement: Multimedia component 4 [file mmc4.docx]

**Supplementary Table S1.** Specific oligonucleotides used in PCR and sequencing analyses.

| Target | Sequence 5' → 3' | |  |
| --- | --- | --- | --- |
|  | **Forward** | **Reverse** | **Amplicon size (bp)** |
| *gltA* (PCR) | GAG AGA AAA TTA TAT CCA AAT GTT GAT | AGG GTC TTC GTG CAT TTC TT | 147 |
| *gltA* (Sequencing) | GCA AGT ATC GGT GAG GAT GTA AT | GCT TCC TTA AAA TTC AAT AAA TCA GGA T | 401 |
| *rickA* (Sequencing) | GGC AAA ATG TTA AAA ATG TTT | CCR GYT TTT TAA CCG TAG TAG | 750 |
